# Effect of Rhythmic Auditory Stimulation on Gait in Parkinsonian Patients with and without Freezing of Gait

**DOI:** 10.1371/journal.pone.0009675

**Published:** 2010-03-22

**Authors:** Pablo Arias, Javier Cudeiro

**Affiliations:** Neuroscience and Motor Control Group (NEUROcom), Department of Medicine-INEF, University of A Coruña, A Coruña, Spain; Baylor College of Medicine, United States of America

## Abstract

Freezing of gait (FOG) in Parkinson's disease (PD) rises in prevalence when the effect of medications decays. It is known that auditory rhythmic stimulation improves gait in patients without FOG (PD-FOG), but its putative effect on patients with FOG (PD+FOG) at the end of dose has not been evaluated yet. This work evaluates the effect of auditory rhythmic stimulation on PD+FOG at the end of dose. 10 PD+FOG and 9 PD-FOG patients both at the end of dose periods, and 10 healthy controls were asked to perform several walking tasks. Tasks were performed in the presence and absence of auditory sensory stimulation. All PD+FOG suffered FOG during the task. The presence of auditory rhythmic stimulation (10% above preferred walking cadence) led PD+FOG to significantly reduce FOG. Velocity and cadence were increased, and turn time reduced in all groups. We conclude that auditory stimulation at the frequency proposed may be useful to avoid freezing episodes in PD+FOG.

## Introduction

The gait of people with Parkinson's disease (PD) is characterised by a number of well-defined features. From a kinematic point of view PD exhibit a reduction in step length and velocity [1]-[3], decreased angular displacement and velocity of lower and upper limbs [4], high stride cycle time variability [5], [6], poor bilateral coordination [7] or asymmetric leg function [8]; and difficult in turning (displaying a block-like pattern [9]). There are other less frequent gait disturbances in PD, among which freezing of gait (FOG) is one of the most disabling. Of unknown origin, FOG is characterised by a sudden loss of the ability to start or continue walking, as if the patient's feet were glued to the ground, which can lead to falls and injuries [10].

FOG is typical in advanced phases of the disease and it seems associated with disease duration, its grade of development, longer duration of levodopa treatment, levodopa-induced dyskinesias [11], [12], as well as early morning dystonia and postural instability [12].

Three main forms of FOG have been identified [13]: a purely akinetic form; a “tremble in place” type at which the patients' legs can tremble between 2–4 Hz [14], and a “shuffling” form with small steps.

Based on the poor correlation between FOG and UPDRS sub-scores [15] has been suggested that FOG has a different origin when compared to other clinical features, such as rigidity or bradykinesia. Also, the EMG profile prior to freezing has shown an altered premature discharge pattern in antagonist leg muscles [16]. This feature may be related to the reported increase in the CV_stride-time_ in advanced PD [6], [17], the poor bilateral coordination [7] and asymmetric motor function in patients suffering FOG [8]. All these characteristics support that loss of control of the regulation of cadence brings about FOG [18].

FOG is chiefly triggered at onset of walking and during turning, but also at narrow spaces (such as doorways) (see supporting information multimedia files Videos S1 and S3) or when approaching targets [11], [19]; its duration is usually less than 10 sec, and rarely longer than 30 sec [13], [19]. Administration of L-dopa can reduce FOG [19], which is more common when medication wears off, suggesting dopamine deficiency as a cause [10], although pedunculopontine nucleus degeneration may also have a role [20], [21].

Although the effect of auditory rhythmic cueing on gait in PD is well documented [6], [22]–[27], reports assessing its role on FOG are much scarcer. Lack of effect on FOG (or even worsening) was reported using auditory [28], [29], as well as other cueing strategies (visual-spatial stimulation [30]). In addition Enzensberger and Fischer [31] have found a significant reduction in the number of freezing episodes at turning and on straight walking in Parkinsonian patients ON-dose while using auditory stimulation at a fixed metronome frequency for all patients. Our objective is to determine the impact of auditory stimulation on FOG when the effect of the medication has decayed, but importantly using a frequency of stimulation normalised as a function of gait pattern of each patient.

## Methods

### Objectives

The aim of our study was to investigate the effect of rhythmic auditory stimulation on the gait of Parkinsonian patients who exhibit significant FOG (PD+FOG) during their *end of dose-periods*. Based on previous results [6], [32], the frequency of stimulation was set to 110% of the *ON-periods* cadence for each subject during preferred walking. Stimulation at this frequency is known to reduce the CV of stride time [6], [32] which is associated with FOG [17]. The research hypothesis is that auditory stimulation at the frequency proposed modifies the walking pattern in PD+FOG, reducing the freezing episodes.

### Participants

Participants in the study were recruited from a total of 80 patients belonging to the Asociación Parkinson Galicia and the Asociación Parkinson Ferrol (Spain). All patients were only orally medicated, without surgical operation for PD.

#### PD+FOG

Patients in this group, who exhibited significant FOG (PD+FOG), had to match the following criteria:

- diagnosis of idiopathic PD based on the UK Parkinson's Disease Society Brain Bank for clinical diagnostic criteria
- history of freezing during walking from medical records, and score >10 (all PD displaying ≥2 in item #3) in the *Freezing of Gait Questionnaire* (FOG*Q*) [33]
- predictable motor fluctuation related to dose intake, determined from medical records and examination by a neurologist
- lack of auditory-visual impairment, musculoskeletal injury, and MMSE score >24
- at the moment of testing, during the end of dose period, they should be able to walk 6m unaided, turn around and come back despite the freezing episodes, which should be present during preferred walking condition (un-cued, at the end of dose)
- during ON periods they should be able to walk without freezing

10 volunteer PD+FOG matched the criteria and underwent the experimental protocol (6 males, 4 females; 68.20 yrs (±8.03), trochanteral height 0.89 m (±0.06), FOG*Q* score 16.70 (±4.81). Patients did not expect any benefit in their gait patterns from the cues, as their use was explained to be a method to characterize gait. No patient had previous experience on gait cueing.

#### PD-FOG

9 volunteer PD, without history of FOG (PD-FOG), were also recruited (6 males, 3 females; 64.44 yrs (±9.50), trochanteral height 0.88 m (±0.04). Inclusion criteria were the same as stated for PD+FOG, with the exception of those criteria related to FOG. The score in the FOG*Q* had to be zero to be a possible subject in this group.

#### CONTROL SUBJECTS

10 healthy subjects (people from our institution and relatives) were selected as the Control group (8 males, 2 females; 70.20 yrs (±6.84), trochanteral height 0.89 m (±0.04); they were also screened for gait or balance impairment.

### Description of Procedures

Subjects were asked to walk along a corridor (with a door in the middle), touch a button on the wall at the end, turn around, come back and touch the button on the other wall, this task in conception and distance included FOG evoking elements.

Patients came to our laboratory on two consecutive days. The first day they undertook MMSE, UPDRS*_on_* and the first two trials (Baseline) at their preferred walking pattern without the door in the middle of the corridor in order to determine baseline cadence, for which the turn was excluded; all this was carried out during patients' *ON-periods* (after patients confirmed to be in *ON* [34], and under observation by a neurologist).

During the next day, at the end of dose, patients performed the UPDRS-III and 4 trials (2 at their preferred walking without auditory stimulation (PW) and 2 with the stimulation at a frequency 10% faster than the cadence at baseline (110A), both with the door in the middle of the corridor); healthy controls performed the 6 trials in the same day. End of dose was defined as “*deterioration and recurrence of parkinsonian symptoms as a result of shorter (sometimes only 1 to 2 hours) duration of benefit after a given dose of L-dopa.*” [35]. Trials were performed in this sequence to avoid stimulation carryover effect (i.e. the effect that cueing in one trial might have on a subsequent un-cued trial) which has been reported in the literature [22]. In this paradigm we used a frequency of stimulation determined during the ON period, to be used at the end of dose time epoch. This method is supported by previous work showing an effect of medication on stride time variability but not in cadence either in PD+FOG and PD-FOG [17]. This work also showed that cadence is not different between PD+FOG and PD-FOG [17], regardless of the medication state.

**Figure 1:**
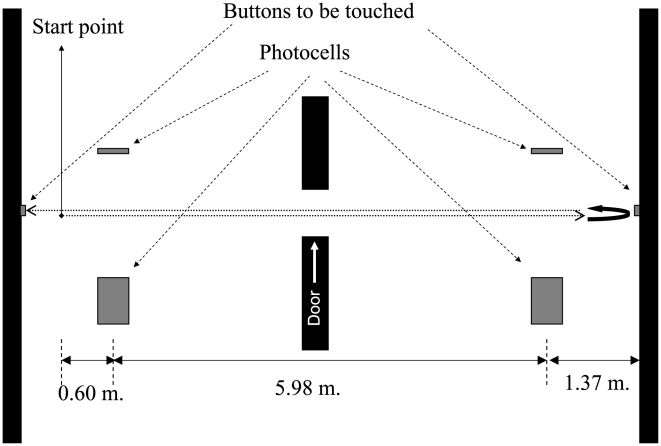
Representation of the task carried-out by the subjects.

The instruction given to the subjects was *“walk along the corridor as you normally do, touch the button on that wall and without stopping turn around, come back, and touch this other button on this wall”* (figure 1). For the stimulation conditions the instruction given was “do the same as before, but matching your steps to the rhythm”. No specific instruction was given in order to manage turns.

All patients were evaluated in the morning after a light breakfast to avoid interference of possible protein intake at lunch, which could lead to L-dopa absorption problems. At the moment of testing PW and 110A patients confirmed to have lost the effect of medication [34]; wearing-off was confirmed by the neurologist.

#### APPARATUS

The recording system consisted of a series of footswitches worn as insoles in the shoes. The footswitches were connected to a radio-transmitter attached to the subjects' belt. Data (sampled at 1KHz) were sent to a receiver unit connected to the computer. This configuration allowed the stride cycle time to be registered.

Two photocells, placed 5.98 m apart, were connected to the recording system so that the records from the moment subjects crossed them were acquired. A portable in-house device provided auditory stimulation (a click) by means of headphones, which subjects wore regardless of whether or not they were stimulated. The sound was a tone with wave-frequency of 4,625 Hz, and the intensity was adjusted to be clearly perceived by the subjects without being annoying. The stimuli were delivered in pulses of 50 ms and the inter-pulse duration was customized to obtain the desired stimulation frequency.

#### ANALYSED VARIABLES

The number of freezing episodes and their duration were measured by analysis of video footage by a specialist with 10 years experience working in a rehabilitation centre for PD, who was unaware of the protocol. Video samples were analysed by means of video software which allows frame identification (and/or sequencing) by simply keyboard strokes, allowing the identification of freezing start and end, duration and number of FOG episodes. Videos to the specialist were presented in random order and were encoded to avoid any kind of identification during evaluation; sound was off. Freezing episodes were defined following the work by Kompoliti et al. [30]: *“One freezing episode was defined as stop and/or hesitation until the next step was accomplished independently of the number of hesitations in place”*.

In other to characterize FOG, the freezing episodes were grouped by duration (less than 3s; 3–10s; >10s) [19], [33]; and by the circumstances under which they occurred: at turning; at the door; at approaching the button to be touched; at walking start. However when evaluating the effect of stimulation, those categories were no used, and only the duration and number of freezing episodes were considered.

Some other kinematic variables were analysed:

Velocity: Calculated as a function of the time to cover the straight section between the photocells, expressed as m/sec.

Cadence: Obtained from footswitch data corresponding to the straight section of the test, expressed as steps/sec.

Step length: Expressed in m as a function of the velocity and the cadence, again measured only over the straight section.

Turn around time: Time taken from the photocell at the end of the corridor (figure 1), which was activated before and after the turn.

The value for each kinematic variable was the mean obtained from the two trials performed in each condition.

### Ethics

All subjects were informed about the nature of the test and signed consent forms. The protocol was in compliance with the Helsinki declaration and was approved by the University of A Coruña Ethics Committee.

### Statistical methods

A student “t” test for independent samples was used to compare the grade of disability between the groups of patients (UPDRS-III).

One-way ANOVA was used to assess differences in motor behaviour at baseline (PW) between groups of patients and controls, also for demographics. Alternatively, a non-parametric Kruskal-Wallis test, and subsequent Mann-Whitney were performed for those variables not matching normality.

In order to determine the effect of stimulation on the kinematics, a 2x3 ANOVA model with repeated measures was performed. Two factors were defined: (i) within-subjects, (factor cue with 2 levels, PW and auditory stimulation (110A)); and (ii) between-subjects, (factor group, with 3 level PD+FOG, PD-FOG, and Controls). Given the parametric nature of this analysis a Logarithmic Transformation was performed when normality was not assumed (in the case of Turning Time for PD+FOG), so that the variables could be introduced into the analysis. Normality of distribution was assessed by means of one sample KS test.

A one-way Chi-Square (χ^2^) was performed in order to assess differences in proportions of type of freezing episodes. Given the task involved passing through a doorway, approaching a point, and start walking twice each trial, and just one turn, the number of three first types was adjusted by dividing each by two. Number and mean duration of the freezing episodes in the PD+FOG in presence vs. absence of stimulation were assessed by means on non-parametric Wilcoxon test. Significance was set at p≤0.05.

## Results

### Characterization

Differences in the UPDRS motor scores between PD+FOG and PD-FOG were not significant (t(17) = 1.163 p = 0.261); proving groups of patients were comparable in the overall disease development, (though clearly they differed in respect of the presence of FOG); demographics were not different along groups p>0.05 (age: F(2,26) = 1.305 p = 0.288; trochanteral height: F(2,26) = 0.029 p = 0.972).

During straight walking, gait patterns exhibited some other characteristic differences between groups (Table 1). One-way ANOVA showed a main effect of the group for velocity and step length. Subsequently pos-hoc analysis showed PD+FOG walked slower, with shorter steps, than PD-FOG and than Controls; this was also seen in PD-FOG vs. Control. The same pattern was displayed for the time to turn as proved by Kruskall Wallis and subsequent Mann-Whitney tests. One-way ANOVA showed, however, that cadence was not difference across groups.

**Table 1:** Characterization of gait kinematics for PD groups and Control in absence of stimulation.

	PD+FOG	PD-FOG	Control		F-p values//KW	
				*PD+FOG vs.PD-FOG*	*PD+FOG vs. Control*	*PD-FOG vs. Control*
**Velocity (m/s)**	0.580 (±0.313)	0.967 (±0.214)	1.237 (±0.160)		F(2,26) = 19.115 p<0.001	
				*p = 0.002*	*p<0.001*	*p = 0.021*
**Step length (m)**	0.337 (±0.174)	0.531 (±0.079)	0.674 (±0.061)		F(2,26) = 20.711 p<0.001	
				*p = 0.001*	*p<0.001*	*p = 0.001*
**Cadence (steps/s)**	1.727 (±0.338)	1.819 (±0.185)	1.831 (±0.125)		F(2,26) = 0.573 p = 0.571	
**Turning Time (s)**	26.886 (±58.690)	3.850 (±1.083)	2.493 (±0.557)		χ^2^ (2) = 18.796 p<0.001	
	*7.515 [3.45–193.30]*	*3.934 [1.81–5.00]*	*2.397 [1.63–3.49]*	*p = 0.004*	*p<0.001*	*p = 0.009*

Values: Mean, (±sd), *median, [range].* Units: (m/s) = meters/second; (m) = meter; (steps/s) = steps/second; (s) = seconds; (n.s) = not significant. Median and range are shown for variables not matching normality. F value for One-Way ANOVA is reported, along with comparisons between groups when significant effect is displayed. For Turning Time, given its non-parametric nature KW test was performed, and subsequent Mann-Whitney test to compare difference between groups. Velocity, step length and turning time were impaired in PD with regards to Controls, and also PD+FOG presented greater degree of impairment than PD-FOG.

**Figure 2:**
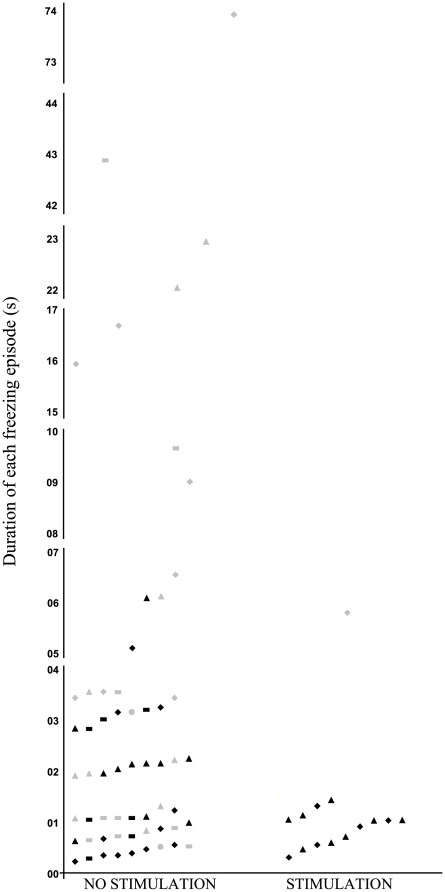
Number and duration of the motor blocks experienced by the patients during walking in absence and presence of stimulation. (▴) At start walking; (♦) at turning; (---) at the door; (•) at approaching the target. Grey icons represent the values obtained for PD+FOG #3 and #10. The number and mean duration of the freezing episodes were significantly reduced by the presence of the stimulation when all PD were analysed (p = 0.014, and p = 0.017; respectively). When PD+FOG #3 & #10 were excluded from the analysis, in order to know if change was due to behaviour of these two extreme PD+FOG, the effect of stimulation kept on being significant, by reducing the number (p = 0.040) and mean duration (p = 0.050) of motor blocks.

All the PD+FOG experienced freezing during the task. A total of 59 freezing episodes (see Table 2 and Fig. 2) were registered during gait without stimulation. Most of them occurred when turning and start hesitation, but also at the door and approaching a target (20, 21, 16, and 2 respectively; χ^2^(3) = 18.75 p<0.001). Only 6 freezing episodes lasted more than 10 sec; 16 lasted between 3–10 sec; and 37 lasted less than 3 sec (χ^2^(2) = 25.46 p≤0.001).

**Table 2:** Effect of the stimulation on walking parameters.

	PW	110A	ANOVA: Factor Cue (C); Factor group (G); and their interaction (C*G)
**Velocity (m/s)**	0.927 (±0.361)	1.008 (±0.328)	**C**: F(1,26) = 8.437 **p = 0.007** C*G: F(2,26) = 1.845 p = 0.178 **G**: F(2,26) = 19.050 **p<0.001**
**Step length (m)**	0.513 (±0.182)	0.532 (±0.154)	**C**: F(1,26) = 1.842 p = 0.186 C*G: F(2,26) = 2.451 p = 0.106 **G**: F(2,26) = 21,592 **p<0.001**
**Cadence (steps/s)**	1.792 (±0.232)	1.878 (±0.184)	**C**: F(1,26) = 5.857 **p = 0.023** C*G: F(2,26) = 0.283 p = 0.756 **G**: F(2,26) = 0.619 p = 0.546
**Turning Time (s)**	11.325 (±35.212)	4.048 (±2.500)	**C**: F(1,26) = 4.882 **p = 0.036** C*G: F(2,26) = 2.255 p = 0.125) **G**: F(2,26) = 13.537 **p<0.001**

	PW	110A	Wilcoxon
**Number of freezing episodes in PD+FOG**	5.900 (±6.707) *2.500 [1–22.00]*	1.400 (±1.265) *1.000 [0–3.00]*	Z = 2.446; **p = 0.014**
**Mean duration of freezing episodes (s) in PD+FOG**	3.119 (±4.930) *0.910 [0.28–16.01]*	1.020 (±1.699) *0.665 [0–5.71]*	Z = 2.395 **p = 0.017**

PW (absence of stimulation); 110A (presence of stimulation). Values: Mean, (±sd), *median, [range]*. Units: (m/s) = meters/second; (m) = meter; (steps/s) = steps/second; (s) = seconds. Results for velocity, step length, cadence and turning time are shown pooled across groups because ANOVA showed lack of significant interaction cue*group, meaning all groups were affected in the same way. Log transformations were applied to Turning Time in order to make distributions adjusted to normality so that making parametric analysis applicable, its mean and (±sd) are plotted without transformation to make interpretation feasible. The stimulation led to increased velocity and cadence, and also to reduce the time to turn, which was seen in the three groups.

Variables related to freezing are only related to PD+FOG, median and range are shown as variables were not matching normality. Stimulation led both to reduce the number and the mean duration of the freezing episodes.

### Effect of the stimulation on gait patterns

**Figure 3:**
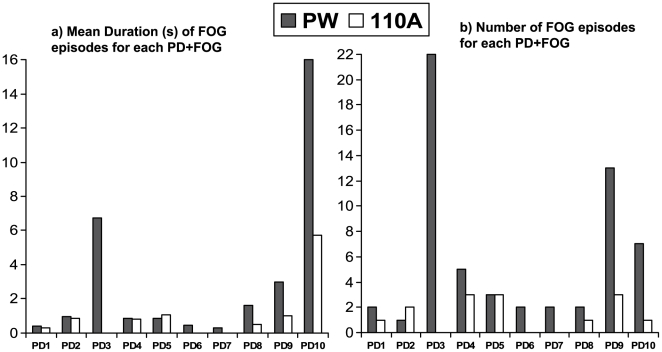
Effect of the stimulation on gait. Mean duration (a), and number (b) of the freezing episodes for each patient. PW (absence of stimulation); 110A (presence of stimulation).

The main outcome of this study is that the number of freezing episodes were significantly reduced in patients in presence of auditory stimulation, from 59 to 14 (6 when turning and 8 at start walking; Z = 2.446 p = 0.014). Mean duration was also significantly reduced (Z = 2.395 p = 0.017); see Table 2, Fig. 2, and supporting multimedia files, Videos S1, S2, S3 and S4. Individual changes in the number and duration of freezing episodes are shown in Fig. 3.

It is possible that the significant reduction in the number and duration of FOG is due to change in very few subjects, rather than to the whole population. For example, PD+FOG subjects number 3 and number 10 (Fig. 3) display considerable more FOG episodes, with longer duration. To assess this, the effect of the stimulation was also checked leaving out PD+FOG #3 and PD+FOG #10. When analysed this way the effect of stimulation remained significant for both reduction in number (Z = 2.058 p = 0.040) and mean duration of motor blocks (Z = 1.960 p = 0.050).

For the rest of variables, stimulation proved to affect the same way all groups, as demonstrated by the lack of significant interactions cue*group. Taking this into account the stimulation led to reduce the time to turn, to increase cadence, and to increase velocity, as proved by a main effect of factor cue for each of those kinematics (see table 2). The increase in step length did not reach significance, however. Significance of factor group for velocity, step length, and turn time proved groups kept on being different along conditions, this was expectable given they were different at PW and they were also equally affected by the stimulation (all main effects and interactions are reported in table 2).

## Discussion

In absence of auditory stimulation the gait of the Parkinsonian patients who “freeze” compared to those without freezing, and the latter compared to controls, exhibited lower velocity, and shorter step length, and such differences from Controls are in agreement with previous work [26]. All PD+FOG also suffered freezing episodes and they took longer to complete turns (vs. PD-FOG; and Controls), PD-FOG also took longer than Controls [36].

However, the main outcome of our study is that auditory stimulation at the frequency proposed significantly reduces the number and the mean duration of the freezing episodes in a FOG eliciting task, aimed to reproduce daily activities [11], and evaluated when the effect of medication decayed (a critical time for patients). Clearly, the effect of the stimulation in reducing FOG is not only driven for some small sample of PD+FOG, but instead included an overall group improvement, since the results are consistent when all patient were analysed and when we excluded those with the highest improvement in presence of stimulation. Importantly, calculating the stimulation frequency during the ON period (FOG free condition) to be used when in the OFF period is an approach not used before. This has turned out to be useful and would be feasible for daily use.

The reduction in FOG is in contrast to a previous study which reported a lack of effect of auditory stimulation on FOG [28]. It seems likely that stimulation frequency plays an important role here, as Cubo et al. [28] utilised a frequency equalling PW cadence, while we used 110% of preferred walking. The CV of stride time is strongly associated with FOG [17], therefore we decided to use a frequency which has proved to reduced CV_stride-time_ [6], [32]; avoiding those which increase it [6], [23], [26]; also Hausdorff et al. [32] have recently reported no effect on stride time variability when the frequency of the auditory stimulation matches PW cadence, the same frequency used by Cubo et al. [28]. The role of the stimulation frequency seems also reinforced by Moreau et al. [29] who proved higher auditory frequencies (20 and 40% above PW cadence) increased FOG in PD. This is important since Moreau's frequencies are much higher than those reported to reduced CV of stride cycle time [6], [32]. Also, our protocol of stimulation is different to other studies [31] because we normalised the frequency for each subject to +10% PW-cadence and they selected a fixed frequency which turned out to be slightly higher (group mean) than PW-cadence. Additionally, contrary to other published studies [26], [28], [31] [36], our work was carried out on patients at the end of dose periods (when FOG prevalence is higher [19]), rather than during the ON periods, which might also help to explain the contrary results, as ON-freezing is resistant to other therapeutic approaches [30], [37]. It is worth saying that other stimulation frequency has been proposed for freezers during ON-periods [26].

The presence of the auditory stimulation interacted with the kinematic variables the same way across our different groups of subjects [6]. In our study auditory stimulation produced an increase in velocity in all groups [26], [31]. Interestingly, in agreement with others [24], this increase in velocity is chiefly a result of augmentation of cadence but not in step length, which probably reflects an adaptation in the stride pattern made to, for instance, prepare turns, so that explaining why the enlargement in step length is not significant, in contrast to reports with larger walkways and without turns [6], [27], [31]. Also, reduction in turning time fits well with the reduction in the freezing episodes, with a great prevalence during turning, and reinforces the suitability of this form of stimulation in order to improve quality of life in the PD.

### Limitations

Despite our results some questions about the effectiveness of cueing on FOG are still open. Here, the impact of stimulation was assessed for limited period of time, so it is pertinent to ask about its effectiveness during repeated, daily use, give the possibility of habituation to stimulation. Some work has reported rhythmic auditory stimulation entrainment in PD after a programme of auditory stimulation, modifying EMG patterns during gait [38], kinematics [27], [39], and brain activity at rest [39] in patients without freezing, but it has not been consistently explored in PD+FOG. Further, we have used a frequency which has been reported to reduce stride time variability [6], [32], but it could be that other frequencies might also have an impact in FOG, this should be explored, as well as the effect of cueing on PD+FOG during dual-task, which has been reported to reduce attentional cost in the case of PD-FOG when stimulation was mainly auditory [40], [41]. Also, an effect of learning exhibited during cued trials (always performed after un-cued ones) is one option to explain improvement, however in our opinion this can hardly account for the effects we report, given that gait is already a well-learned movement; appropriate randomization of a large enough set of trials could control for the sequence effects, but carry-over effects may also appear [22]. In addition, the protocol might also become too heavy for the patients. In this work we have not assessed the impact of the stimulation on CV of stride cycle time, which has been related to FOG [17]. We deem larger straight trajectories would be needed for this, conversely to the shorter trajectory used in our study (with turns, door…), which was aimed to reproduce FOG eliciting elements.

### Conclusion

We conclude that auditory stimulation may be used in order to minimize FOG at the end of dose in affected Parkinsonian patients. Results from our study support the use of a frequency slightly above the preferred walking frequency (as measured during ON-periods in absence of FOG), which can then be used at the end of dose phase. This point strongly supports other work on the suitability of using auditory cues to improve quality of life in PD either in controlled or uncontrolled environments [42], [43].

## Supporting Information

Video S1 Example of a patient (Example1) with motor blocks (mainly at turning) during preferred walking (no stimulation).
(7.39 MB MPG)

Video S2 Example of the same patient shown in S1 (Example1) with motor blocks during auditory stimulation. Walking and turning were clearly improved.
(2.56 MB MPG)

Video S3 Example of another patient (Example2) with motor blocks (mainly at crossing the door) during preferred walking (no stimulation).
(9.21 MB MPG)

Video S4 Example of the same patient showed in V3 (Example2) with motor blocks (mainly at crossing the door) during auditory stimulation. Walking through the door was clearly improved.
(5.70 MB MPG)
